# Supplementary material for: Ocean-bottom and surface seismometers reveal continuous glacial tremor and slip
Source: Nat Commun. 2021 Jun 24;12:3929. doi: 10.1038/s41467-021-24142-4 (PMC8225613; doi:10.1038/s41467-021-24142-4)
Supplement: Supplementary file 1 — Supplementary Information [file 41467_2021_24142_MOESM1_ESM.pdf]

Ocean-bottom and surface seismometers reveal continuous glacial tremor and slip

Evgeny A. Podolskiy [a,b,*] Yoshio Murai [c], Naoya Kanna [d], and Shin Sugiyama [e,a,b]

[a]Arctic Research Center, Hokkaido University, Sapporo, Japan

[b]Graduate School of Environmental Science, Hokkaido University, Sapporo, Japan

[c]Institute of Seismology and Volcanology, Faculty of Science, Hokkaido University, Sapporo, Japan

[d]Atmosphere and Ocean Research Institute, University of Tokyo, Kashiwa, Japan

[e]Institute of Low Temperature Science, Hokkaido University, Sapporo, Japan

[*]e-mail: evgeniy.podolskiy@gmail.com

Supplementary Figures (1-9)

Supplementary Table (1)

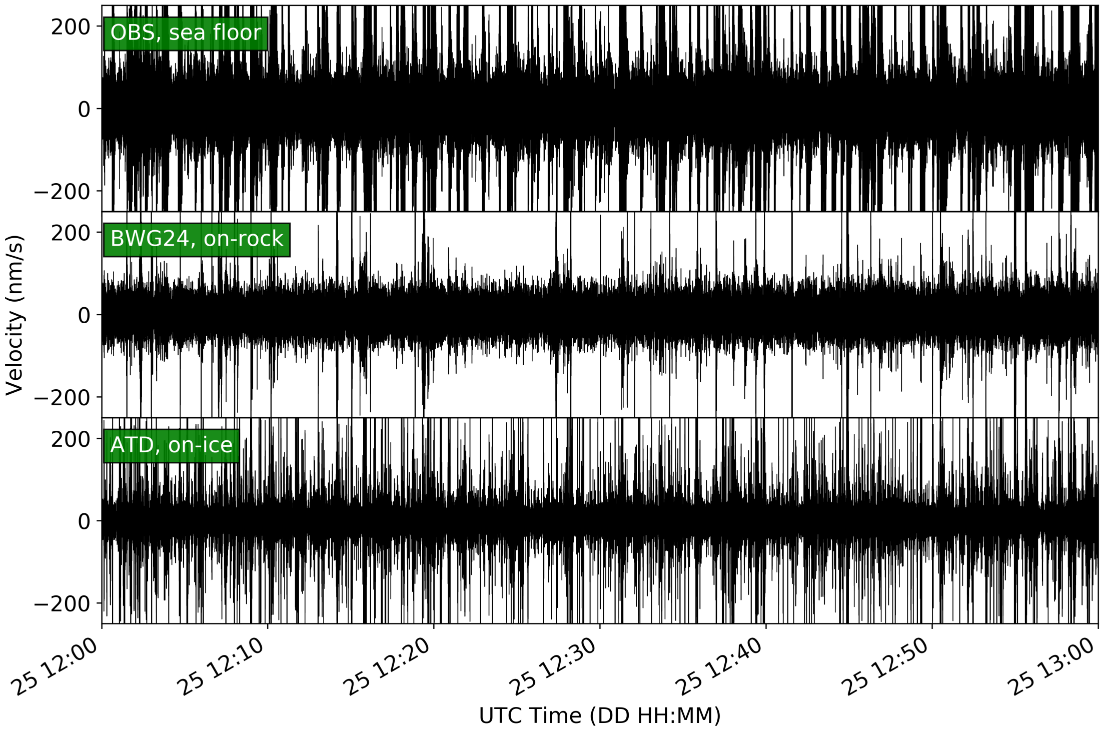

Supplementary Fig. 1: Example of analysed waveforms, 2019. A 1-h-long example of bandpass-filtered (3.5–7.0 Hz) waveforms recorded by three seismic stations underwater, on rock, and on ice (OBS, BWG24, and ATD, respectively) during low-wind conditions (Supplementary Fig. 2), 25 July 2019.

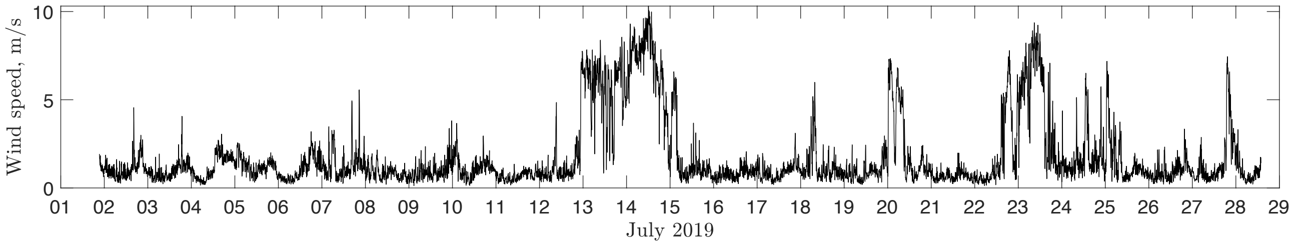

Supplementary Fig. 2: July 2019 wind speed. The wind speed measurements are from an Automatic Weather Station (AWS) near Bowdoin Glacier in July 2019. See Fig. 1b for the AWS location.

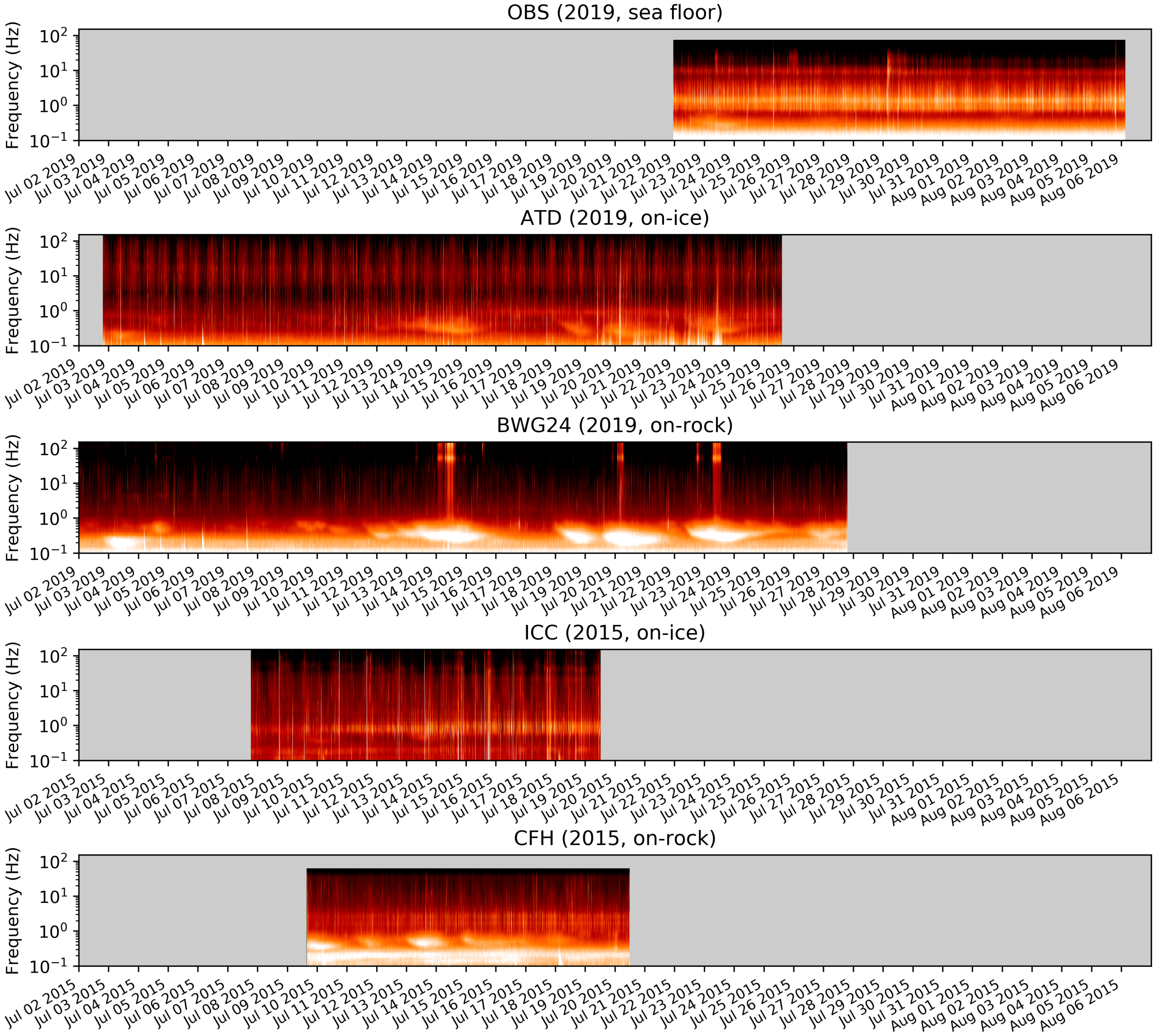

Supplementary Fig. 3: Overview of considered seismic data. Spectrograms of instrument-deconvoluted seismic data (vertical components) computed using the properties of power spectral density--probability density functions (PSD–PDFs) analysis detailed in the main text and Methods[41]. For visual consistency, similar timescales (36 days) and amplitude range (50 dB) are shown for stations recording in 2015 (ICC, CFH) and 2019 (OBS, ATD, BWG24). Previously studied tide-modulated high-frequency seismicity due to crevassing[35,48] is especially pronounced at on-ice stations (ATD and ICC).

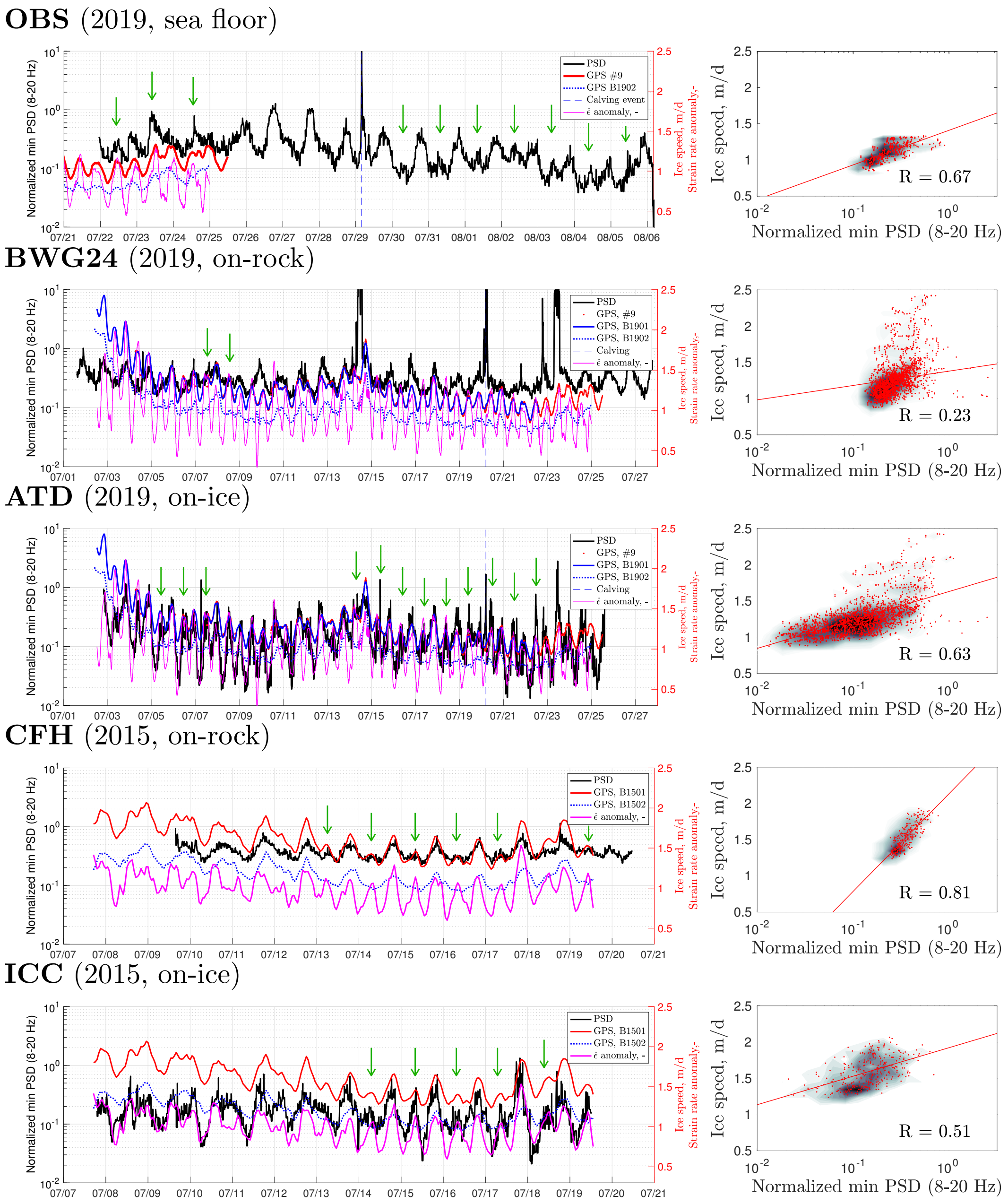

Supplementary Fig. 4: Analysis of high-frequency data from all seismic stations. (Left) Minimum seismic tremor amplitude (8–20.0 Hz power spectral density, PSD) versus glacier displacement rate at Global-Positioning-System (GPS) stations (2019: B1901, B1902, and #9; 2015: B1501 and B1502), with the corresponding strain rate anomaly, $\dot{\varepsilon}$ (i.e., deviation from the mean). The dashed blue line indicates the timing of major calving events (20 and 29 July 2019). Green arrows indicate examples of the morning peaks. (Right) Scatter and density plots showing the correlation between the seismic signal and horizontal GPS displacement rate (at B1901 and B1501 in 2019 and 2015, respectively) for overlapping data (R is the Pearson correlation coefficient). The red line is the regression fit.

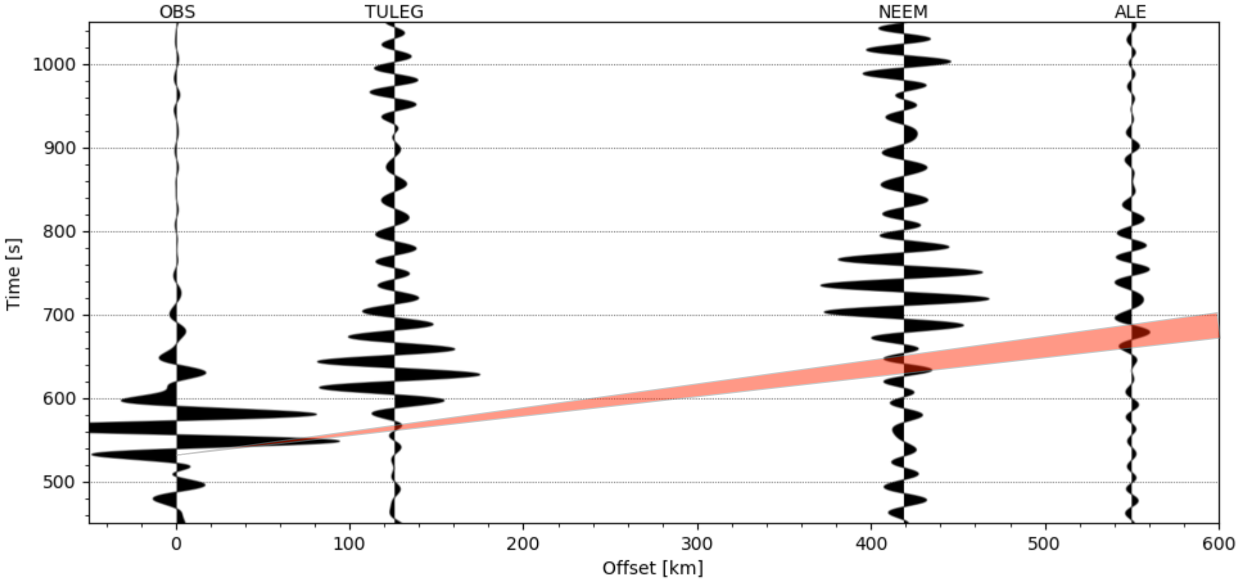

Supplementary Fig. 5: Calving event, 2019. Moveout in waveforms across GLISN stations, filtered between 25 and 50 s. To help the eye, the red wedge indicates a regional surface-wave-velocity range of 3.5–4.25 km s^{-1} (taken after[48, 66]). Time is relative to 03:40 UTC, 29 July 2019.

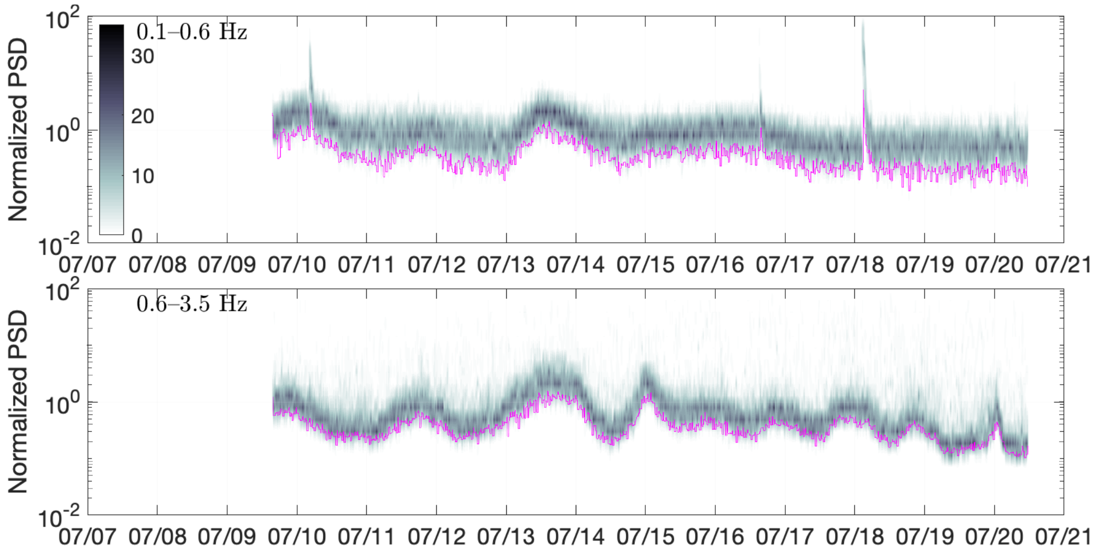

Supplementary Fig. 6: Analysis of data from the 2015 on-rock seismic station (CFH); low frequencies. Density plots of the normalized noise amplitude variations for 0.1–3.5 Hz frequency bands (for velocity power spectral density, PSD). Each magenta curve marks the lowest noise level in a given frequency band.

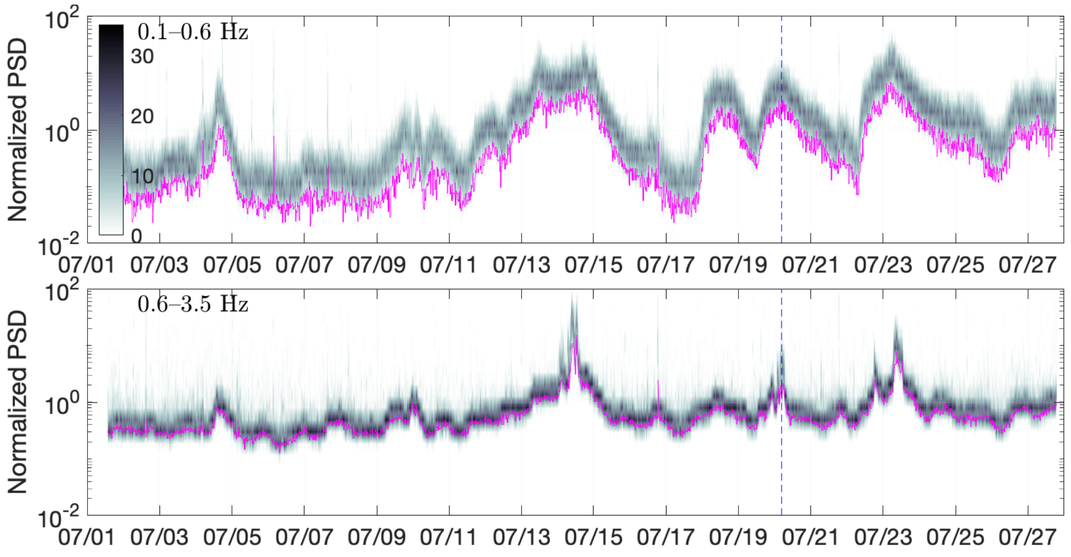

Supplementary Fig. 7: Analysis of data from the 2019 on-rock seismic station (BWG24); low frequencies. Density plots of the normalized noise amplitude variations for 0.1–3.5 Hz frequency bands (for velocity power spectral density, PSD). Each magenta curve marks the lowest noise level in a given frequency band. The dashed blue line indicates the timing of a major calving event.

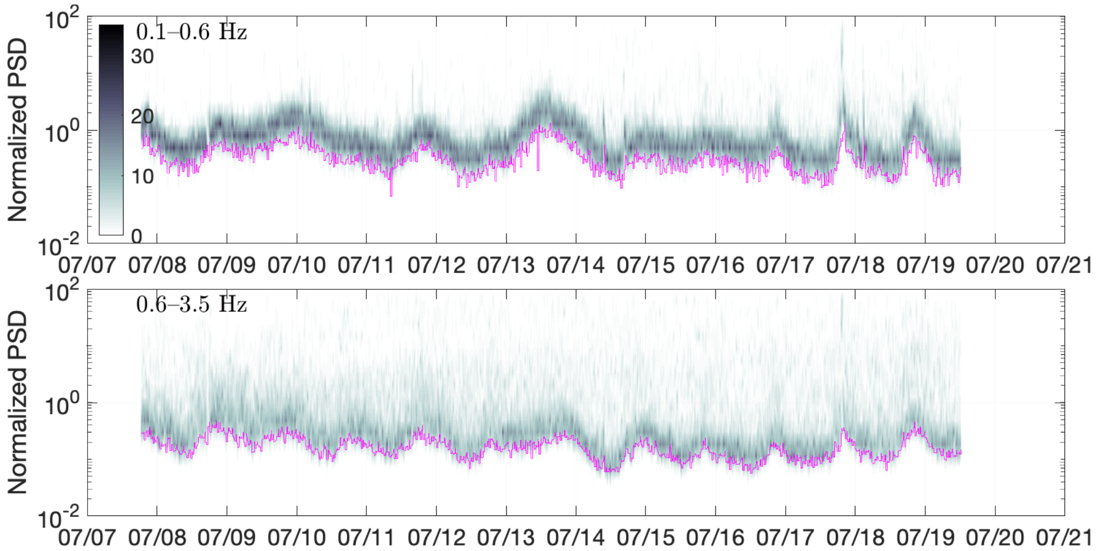

Supplementary Fig. 8: Analysis of data from the 2015 on-ice seismic station (ICC); low frequencies. Density plots of the normalized noise amplitude variations for 0.1–3.5 Hz frequency bands (for velocity power spectral density, PSD). Each magenta curve marks the lowest noise level in a given frequency band.

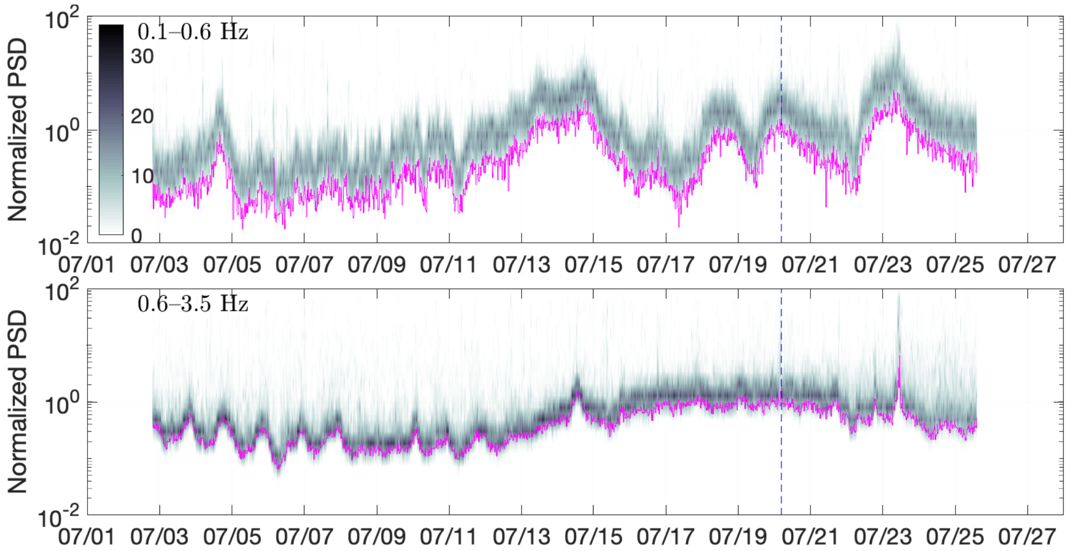

Supplementary Fig. 9: Analysis of data from the 2019 on-ice seismic station (ATD); low frequencies. Density plots of the normalized noise amplitude variations for 0.1–3.5 Hz frequency bands (for velocity power spectral density, PSD). Each magenta curve marks the lowest noise level in a given frequency band. The dashed blue line indicates the timing of a major calving event.

Supplementary Table 1: Correlation between the high-frequency seismic signal (11–20 Hz) and ice speed (v) or strain rate ($\dot{\varepsilon}$).

Station	*v*	$\dot{\varepsilon}$
OBS	0.57	0.60
BWG24	0.28	0.17
ATD	0.64	0.69
CFH	0.80	0.69
ICC	0.42	0.54
