# Supplementary material for: Ocean-bottom and surface seismometers reveal continuous glacial tremor and slip
Source: Nat Commun. 2021 Jun 24;12:3929. doi: 10.1038/s41467-021-24142-4 (PMC8225613; doi:10.1038/s41467-021-24142-4)
Supplement: Supplementary file 3 — Description of Additional Supplementary Files [file 41467_2021_24142_MOESM3_ESM.pdf]

Description of Additional Supplementary Files

File name: Supplementary Dataset 1
Description: Ice speed measurements in July 2019 (GPS B1901).

File name: Supplementary Dataset 2
Description Ice speed measurements in July 2019 (GPS B1902).

File name: Supplementary Dataset 3
Description: Ice speed measurements in July 2019 (GPS #9).

File name: Supplementary Dataset 4
Description: Wind speed measurements in July 2019.

File name: Supplementary Dataset 5
Description: Tidal height measurements in July– August 2015 and July–August 2019 (Pituffik/Thule).

File name: Supplementary Dataset 6
Description: Supplementary Software
