# Supplementary figures and images for: Ocean-bottom and surface seismometers reveal continuous glacial tremor and slip
Source: Nat Commun. 2021 Jun 24;12:3929. doi: 10.1038/s41467-021-24142-4 (PMC8225613; doi:10.1038/s41467-021-24142-4)

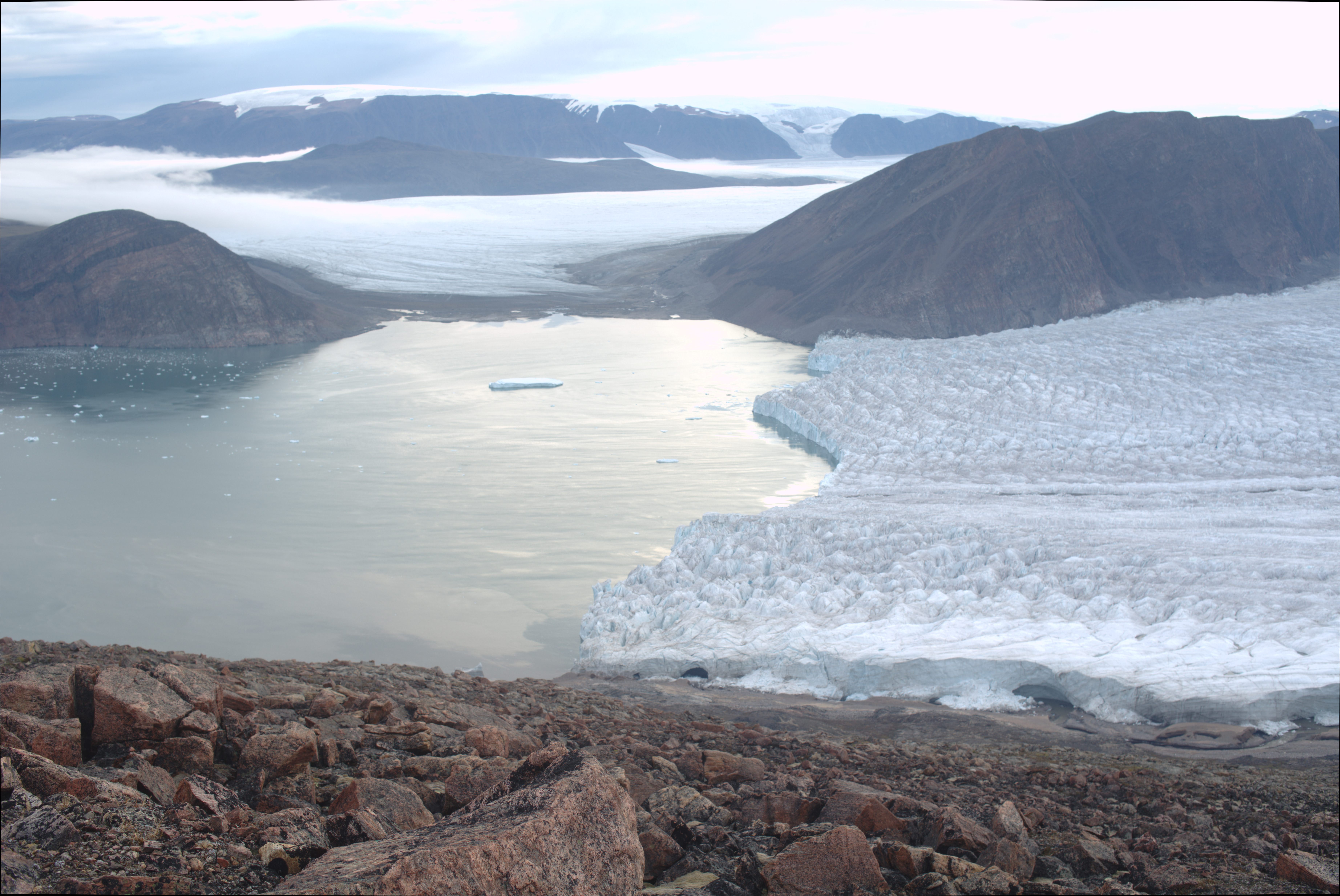

Supplement: Supplementary file 4 — Dataset 6 [file 41467_2021_24142_MOESM4_ESM.zip › code/DSC_2689_03.JPG]

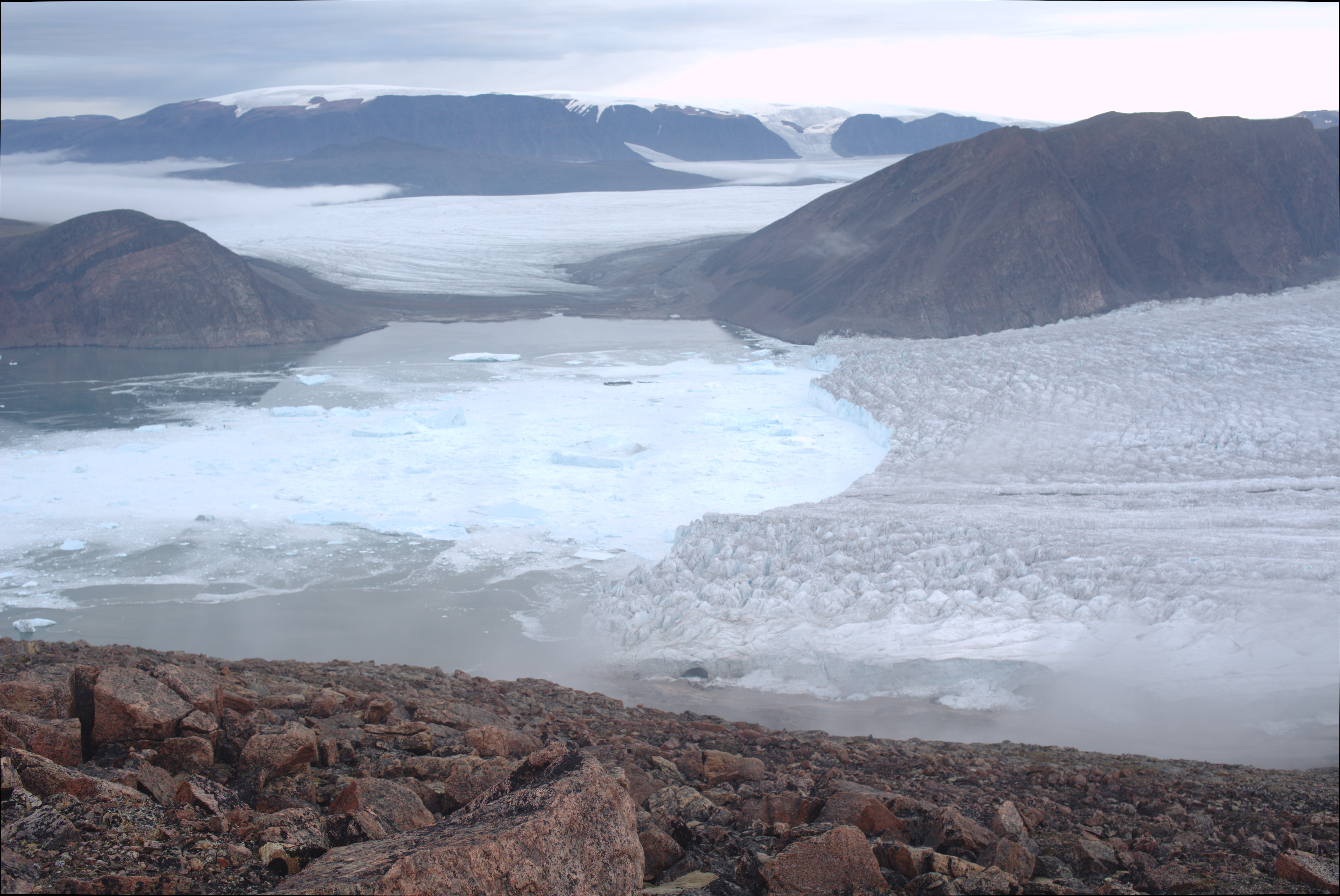

Supplement: Supplementary file 4 — Dataset 6 [file 41467_2021_24142_MOESM4_ESM.zip › code/DSC_2691_05.JPG]

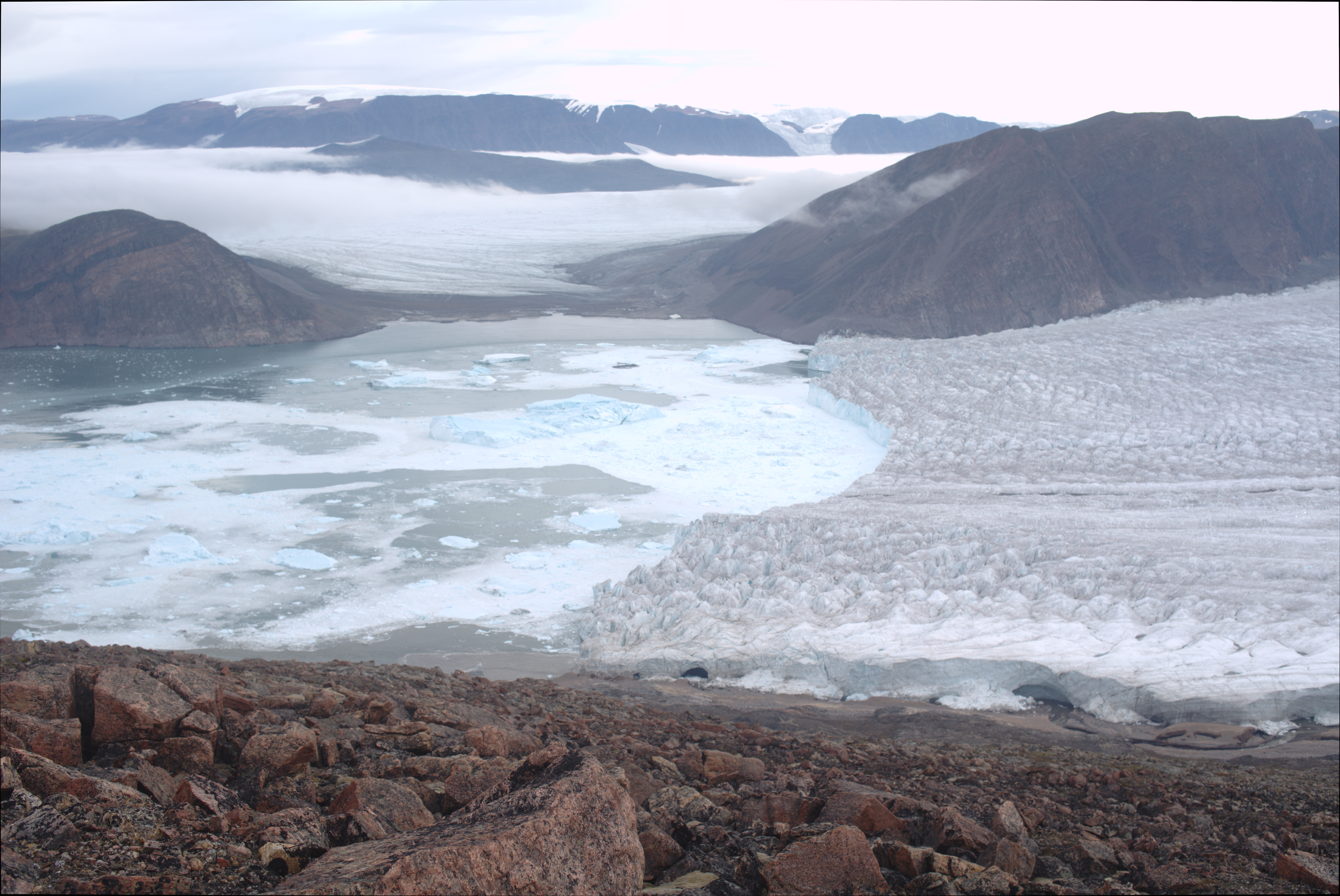

Supplement: Supplementary file 4 — Dataset 6 [file 41467_2021_24142_MOESM4_ESM.zip › code/DSC_2690_04.JPG]
